# A novel risk model for mortality and hospitalization following cardiac resynchronization therapy in patients with non-ischemic cardiomyopathy: the alpha-score

**DOI:** 10.1186/s12872-020-01460-x

**Published:** 2020-04-28

**Authors:** Shengwen Yang, Zhimin Liu, Yiran Hu, Ran Jing, Min Gu, Hongxia Niu, Ligang Ding, Anlu Xing, Shu Zhang, Wei Hua

**Affiliations:** 1 State Key Laboratory of Cardiovascular Disease, Fuwai Hospital, National Center for Cardiovascular Diseases, Chinese Academy of Medical Sciences and Peking Union Medical College, No.167 Beilishi Road, Xicheng District, Beijing, 100037 China; 2 Amazon, Palo alto, CA USA

**Keywords:** Non-ischemic cardiomyopathy, Heart failure, Risk model, Hosmer-Lemeshow test

## Abstract

**Background:**

Non-ischemic cardiomyopathy (NICM) has been associated with a better left ventricle reverse remodeling response and improved clinical outcomes after cardiac resynchronization therapy (CRT). The aims of our study were to identify the predictors of mortality and heart failure hospitalization in patients treated with CRT and design a risk score for prognosis.

**Methods:**

A cohort of 422 consecutive NICM patients with CRT was retrospectively enrolled between January 2010 and December 2017. The primary endpoint was all-cause mortality and heart transplantation.

**Results:**

In a multivariate analysis, the predictors of all-cause death were left atrial diameter [Hazard ratio (HR): 1.056, 95% confidence interval (CI): 1.020–1.093, *P* = 0.002]; non-left bundle branch block [HR: 1.793, 95% CI: 1.131–2.844, *P* = 0.013]; high sensitivity C-reactive protein [HR: 1.081, 95% CI: 1.029–1.134 *P* = 0.002]; and N-terminal pro-B-type natriuretic peptide [HR: 1.018, 95% CI: 1.007–1.030, *P* = 0.002]; and New York Heart Association class IV [HR: 1.018, 95% CI: 1.007–1.030, *P* = 0.002]. The Alpha-score (**A**trial diameter, non-**L**BBB, **P**ro-BNP, **H**s-CRP, NYH**A** class IV) was derived from each independent risk factor. The novel score had good calibration (Hosmer-Lemeshow test, *P* > 0.05) and discrimination for both primary endpoints [c-statistics: 0.749 (95% CI: 0.694–0.804), *P* < 0.001] or heart failure hospitalization [c-statistics: 0.692 (95% CI: 0.639–0.745), *P* < 0.001].

**Conclusion:**

The Alpha-score may enable improved discrimination and accurate prediction of long-term outcomes among NICM patients with CRT.

## Background

Cardiac resynchronization therapy (CRT) improves cardiac function and decreases hospital admissions and mortality among patients with advanced heart failure (HF) and left ventricular dyssynchrony [1–3]. However, based on data derived from numerous large-sample, randomized trials, approximately one-third of all CRT recipients fail to achieve the expected benefit from the device [4]. Since the implantation of CRT devices is an invasive approach with a relatively high the economic burden, the application of a risk model for candidate stratification could aide in the optimal selection of patients and in identifying eligible patients likely to receive the greatest benefit. Non-ischemic cardiomyopathy (NICM) is one of the major causes of HF, especially in Asia [5, 6]. Given that patients with NICM have distinctive pathophysiology compared to that of ischemic patients, their predictors could be different from the that of published models, and the weight of the values for similar predictors may be distinct [7]. Multiple studies have attempted to combine various clinical and biomarker metrics into a risk score to predict the prognosis [8–11]. However, to our knowledge, a predictive risk model for long-term outcomes focused on NICM patients with CRT is lacking.

Therefore, our study focused on (i) investigating the independent predictors of all-cause mortality and heart transplantation or HF in NICM patients treated with CRT; (ii) developing a new risk model for stratifying NICM CRT candidates and assessing its performance for all-cause mortality, heart transplantation, and HF hospitalization in NICM.

## Methods

### Study population

We enrolled 459 consecutive patients with CRT in the Arrhythmia Center of Fuwai Hospital between January 2010 and December 2017.

Diagnosis of NICM patients was made according to classification by the cardiomyopathies’ criteria [12], defined as the presence of systolic dysfunction without a history of myocardial infarction and/or the absence of significant coronary artery disease documented on a coronary angiogram. Inclusion criteria were in accordance with guidelines for cardiac resynchronization and defibrillation [13]: symptomatic HF, left ventricle (LV) ejection fraction < 35% and QRS exhibiting left bundle branch lock with a duration ≥120 ms. All patients received optimal medical therapy for at least 3 months prior to CRT implantation. Patients were excluded if they (1) were aged < 18 years, (2) were pregnant, (3) had prior pacemakers or implantable cardioverter defibrillator implantation, (4) were patients classified as a non-ambulatory New York Heart Association (NYHA) class IV, or (5) lost to follow-up.

**Fig. 1:**
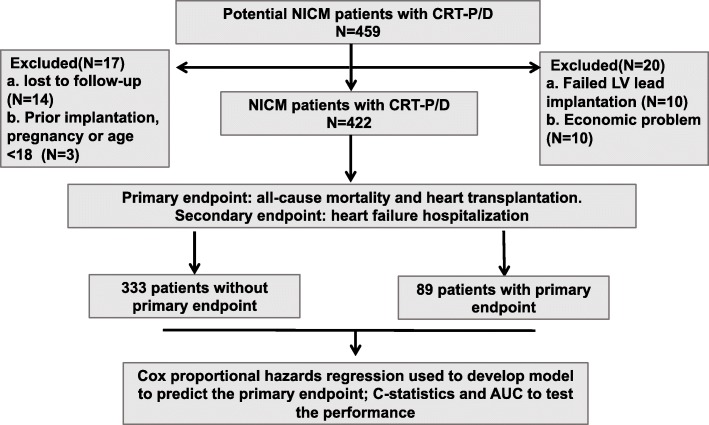
A diagram to describe the flow of participants through the study

Ten candidates failed LV lead implantation; ten declined CRT implantation because of financial difficulties; three patients were excluded based on the exclusion criteria, and 14 patients were lost during follow-up. Finally, a total of 422 eligible NICM patients with CRT were enrolled (Fig. 1).

The Institutional Review Board of Fuwai Hospital approved the study, and all participants provided signed informed consent.

### Device implantation

All patients were implanted with a cardiac resynchronization therapy- pacemaker (CRT-P) or cardiac resynchronization therapy- defibrillator (CRT-D) according to contemporary clinical practice guidelines [13]. The inclusion criteria were: symptomatic HF, LV ejection fraction < 35% and QRS exhibiting left bundle branch lock with a duration ≥120 ms. The leads of the LV were inserted into a lateral or posterolateral coronary sinus branch through the subclavian route. The interventricular and atrioventricular intervals were optimized by program ming on the day after implantation, which allowed maximizing the amount of BiV (as close to 95% as possible) by electrocardiography (ECG). All participants followed up with optimal programming and received standard medications for HF after implantation.

### Follow-up and study endpoints

All patients underwent regular follow-up via outpatient clinical visits or telephone interviews. The primary endpoint of the study was all-cause mortality or heart transplantation. The secondary endpoint was hospitalization due to HF. If patients were hospitalized for HF more than once, only the first hospitalization was counted. Two independent physicians who were blinded to the patients’ clinical data evaluated the endpoints.

### Statistical analysis

Statistical analyses were performed using SPSS version 23 (IBM Corp, Chicago, IL). Continuous data are presented as the mean and standard deviation (SD), and categorical variables are presented as numbers and percentages. The Kolmogorov-Smirnov test was used to test the normality of the distribution of continuous variables. Student’s t-test, nonparametric equivalent tests and chi-square tests were utilized as appropriate. For N-terminal pro-B-type natriuretic peptide (NT pro-BNP), pg/100 ml was used as its unit of measurement. The Kaplan-Meier method was used to construct survival curve with a log-rank test according to the different scores and risk groups. Adjusted hazard ratios were calculated by Cox regression analysis after correcting for differences in baseline characteristics. Variables with a bootstrapped *P* < 0.05 were assigned a weighted point score based on their associated hazard ratio and a simple score was calculated via the addition of all the points. The optimal cutoff point was detected by identifying the Youden index point (sensitivity +specificity – 1). The area under the receiver-operating characteristic curve (AUC) or c statistic to assess the discrimination in receiver operating characteristic (ROC) curves. The calibration of the score was assessed by the Hosmer-Lemeshow test. The AUC ranged from 0.5 (no discrimination) to 1.0 (perfect discrimination). Two-sided *p* values < 0.05 were considered statistically significant.

## Results

### Baseline characteristics

Table 1 lists the baseline characteristics of the study population. The study cohort was comprised of 422 consecutively enrolled NICM patients implanted with CRT-D or CRT-P devices. The mean age of the patients was 59 ± 11 years; 64.7% (273/422) were male; 43.4% (183/422) were implanted with CRT-D, and 70.4% (297/422) exhibited a left bundle branch block (LBBB) on an ECG. During a median follow-up period of 22.82 months (12.17–37.20 months), 89 primary endpoints events occurred: 81 deaths, 10 heart transplantations (including two patients who died after the heart transplantation). There was a total of 113 patients with hospitalizations for HF. Compared with patients without primary endpoint events, patients with primary endpoint events had lower left ventricular ejection fraction (LVEF), higher NT Pro-BNP level, and a decreased use of Angiotensin converting enzyme inhibitors (ACEIs) or angiotensin receptor blockers (ARB) in patients with events; however, there were no statistically statistical differences in age, sex, CRT type, or prevalence of atrial fibrillation at baseline between the two groups.

**Table 1:** Baseline characteristics

Variables	Overall *N* = 422	Patients with primary endpoint events *N* = 333	Patients without primary endpoint events *N* = 89	*P*-value
Age, y	59 ± 11	59 ± 11	58 ± 12	0.989
Male, n (%)	273(64.7)	208(62.5)	65(73.0)	0.064
CRT-D, n (%)	183(43.4)	138(41.4)	45(50.6)	0.123
BMI, (kg/m^2^)	24.53 ± 4.71	24.85 ± 4.93	2323 ± 3.46	0.006
Atrial fibrillation, n (%)	71(16.8)	50(15.0)	21(23.6)	0.055
LBBB, n (%)	297(70.4)	250(75.1)	47(52.8)	< 0.001
NYHA class, n (%)				
II	128(30.3)	113(33.9)	15(16.9)	0.002
III	239(56.6)	187(56.2)	52(58.4)	0.701
IV	55(13)	33(9.9)	22(24.7)	< 0.001
Initial QRS width(ms)	166.92 ± 22.58	164.43 ± 23.44	168.81 ± 19.03	0.065
Echocardiography				
LA(mm)	44.14 ± 7.41	43.38 ± 7.19	47.00 ± 7.54	< 0.001
LVEDD(mm)	70.09 ± 10.69	69.34 ± 10.62	72.91 ± 10.52	0.005
LVEF (%)	30.45 ± 8.45	30.95 ± 8.53	28.60 ± 7.91	0.020
Laboratory factors				
NT-proBNP (pg/ml)	2173 ± 2018	1912 ± 1798	3150 ± 2462	< 0.001
Uric Acid (umol/L)	433.65 ± 130.00	430.15 ± 124.65	446.75 ± 148.35	0.285
HsCRP(mg/L)	2.79 ± 3.89	3.21 ± 3.48	5.67 ± 4.69	< 0.001
Creatinine(umol/L)	90.98 ± 28.69	88.65 ± 23.66	99.68 ± 41.57	0.001
Albumin	42.13 ± 4.87	42.62 ± 4.94	40.31 ± 4.15	< 0.001
AST	23.41 ± 11.88	22.56 ± 10.97	26.55 ± 14.42	0.005
Big endothelin-1	0.55 ± 0.41	0.49 ± 0.37	0.78 ± 0.45	< 0.001
Medications				
ACEI/ARB, n (%)	330(78.2)	273(82.0)	57(64.0)	< 0.001
Beta-blockers, n (%)	384(91)	304(91.3)	80(89.9)	0.681
Diuretics, n (%)	396(93.8)	313(94.0)	83(93.3)	0.798
Spironolactone, n (%)	373(88.4)	299(89.8)	74(83.1)	0.082

*CRT-D* cardiac resynchronization therapy with a defibrillator, *BMI* Body mass index, *LBBB* Left bundle branch block, *RBBB* Right bundle branch block, *NYHA* The New York Heart Association Functional Classification, *LA* Left atrial dimeters, *LVEDD* Left ventricular end diastolic diameter, *LVEF* Left ventricular ejection fraction, *NT-proBNP* N-terminal pro-B-type natriuretic peptide, *HsCRP* High-sensitivity C-reactive protein, *LDL-C* Low density lipoprotein cholesterol, *HDL-C* High density lipoprotein cholesterol, *AST* Aspartate aminotransferase, *ACEI* Angiotensin converting enzyme inhibitor, *ARB* Angiotensin receptor blockers; *P*-value:Comparison between derivation cohort and validation cohort

### Independent predictors of the primary endpoint from the derivation dataset

In the multivariable analysis (Table 2), five independent predictors were associated with the risk of the primary endpoint: left atrium (LA) diameter [Hazard ratio (HR): 1.056, 95% confidence interval (CI): 1.020–1.093, *P* = 0.002]; non-LBBB [HR: 1.793, 95% CI: 1.131–2.844, *P* = 0.013]; high sensitivity C-reactive protein (HsCRP) [HR: 1.081, 95% CI: 1.029–1.134 *P* = 0.002]; and NT Pro-BNP [HR: 1.018, 95% CI: 1.007–1.030, *P* = 0.002]; and New York Heart Association (NYHA) class IV [HR: 1.018, 95% CI: 1.007–1.030, *P* = 0.002].

**Table 2:** Predictors of all-cause mortality and heart transplantation risk by uni- and multivariate Cox proportional hazards

Variables	Univariate		Multivariate	
	HR(95% CI)	*P*-value	HR(95% CI)	*P*-value
Age	0.996(0.977–1.015)	0.667		
gender(male)	1.715(1.072–2.743)	0.024		
Non-LBBB	2.142(1.412–3.248)	< 0.001	1.718(1.128–2.616)	0.012
Type of device (CRT-D)	1.489(0.980–2.260)	0.062		
Atrial Fibrillation	1.748(1.070–2.858)	0.026		
NYHA function class IV	2.356(1.455–3.817)	< 0.001	1.663(1.020–2.712)	0.042
AST	1.018(1.005–1.030)	0.005		
HS-CRP	1.107(1.060–1.156)	< 0.001	1.065(1.018–1.114)	0.006
NT-proBNP per100	1.029(1.021–1.037)	< 0.001	1.018(1.008–1.029)	< 0.001
Big Endothelin-1	1.778(1.256–2.515)	< 0.001		
Creatinine Uric acid	1.008(1.003–1.013) 1.001(1.000–1.003)	0.002 0.063		
LA	1.085(1.054–1.116)	< 0.001	1.052(1.018–1.087)	0.002
LVEDD	1.029(1.010–1.048)	0.003		

Abbreviations as Table 1

We used these five independent predictors: **A**trial diameter, non-**L**BBB, **P**ro-BNP, **H**s-CRP, NYH**A** class IV, to develop the Alpha. Each categorical predictor was assigned 1 point individually. For the continuous parameters, the cutoff points were evaluated by the Youden index point. (Table 3). Score-tertiles were created according to the tertile of the Alpha score (0–1 point as the low-risk group; 2–3 points as the intermediate-risk group, and 4–5 points as the high-risk group).

**Table 3:** The Alpha score standards

Letter	Risk factor	Score (if present)
A	Left atrial diameter (> 44.5 cm)	1
L	non-left bundle branch block	1
P	N-terminal pro-B-type natriuretic peptide (> 13.53 per 100 pg/ml)	1
**H**	high sensitivity C-reactive protein (> 2.87 umol/L)	1
A	NYHA IV	1
Max score		5

### Performance of the alpha-score

**Fig. 2:**
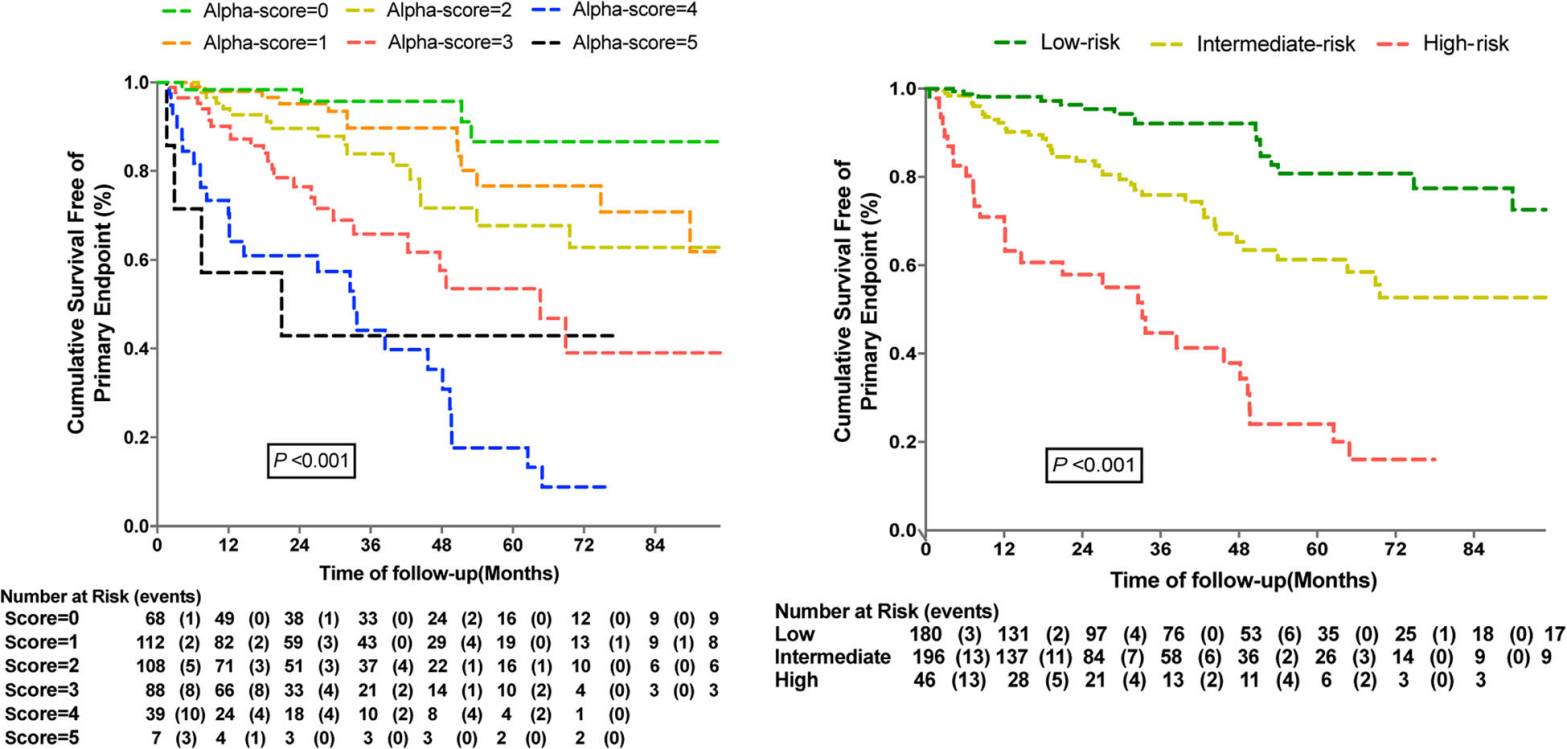
Plot of Kaplan Meier estimates of survival free of primary endpoint according to Alpha-score and score-tertile

**Fig. 3:**
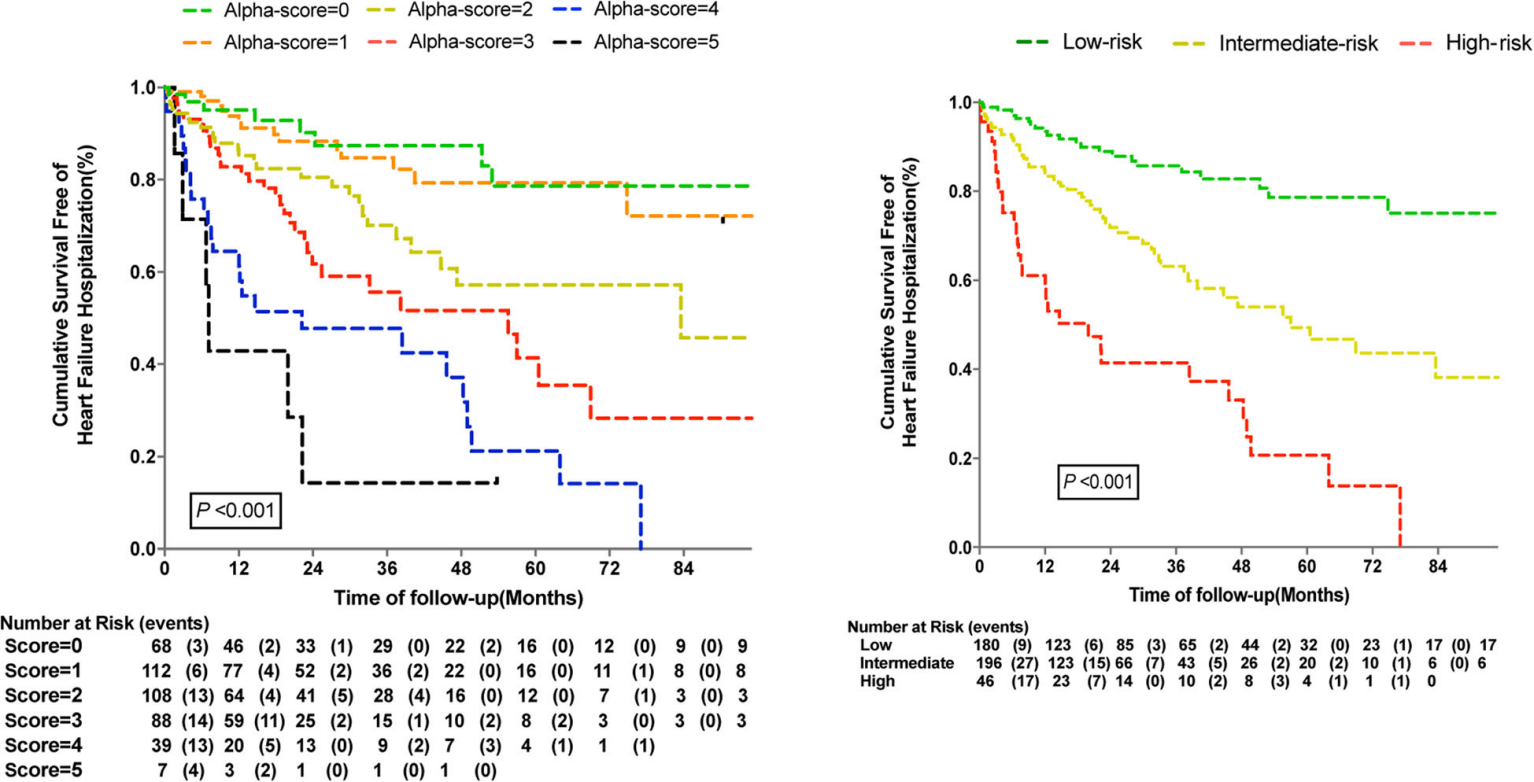
Plot of Kaplan Meier estimates of survival free of heart failure hospitalization according to Alpha-score and score-tertile

As shown in Figs. 2 and 3, the risk of poor outcomes increased with the accumulation of risk factors. Kaplan-Meier survival estimates, according to the Alpha scores and different risk groups for the primary endpoint and HF hospitalization. Notably, based on the Alpha-score system, the rate of HF hospitalization among patients with higher scores was significantly higher than those with lower scores.

**Fig. 4:**
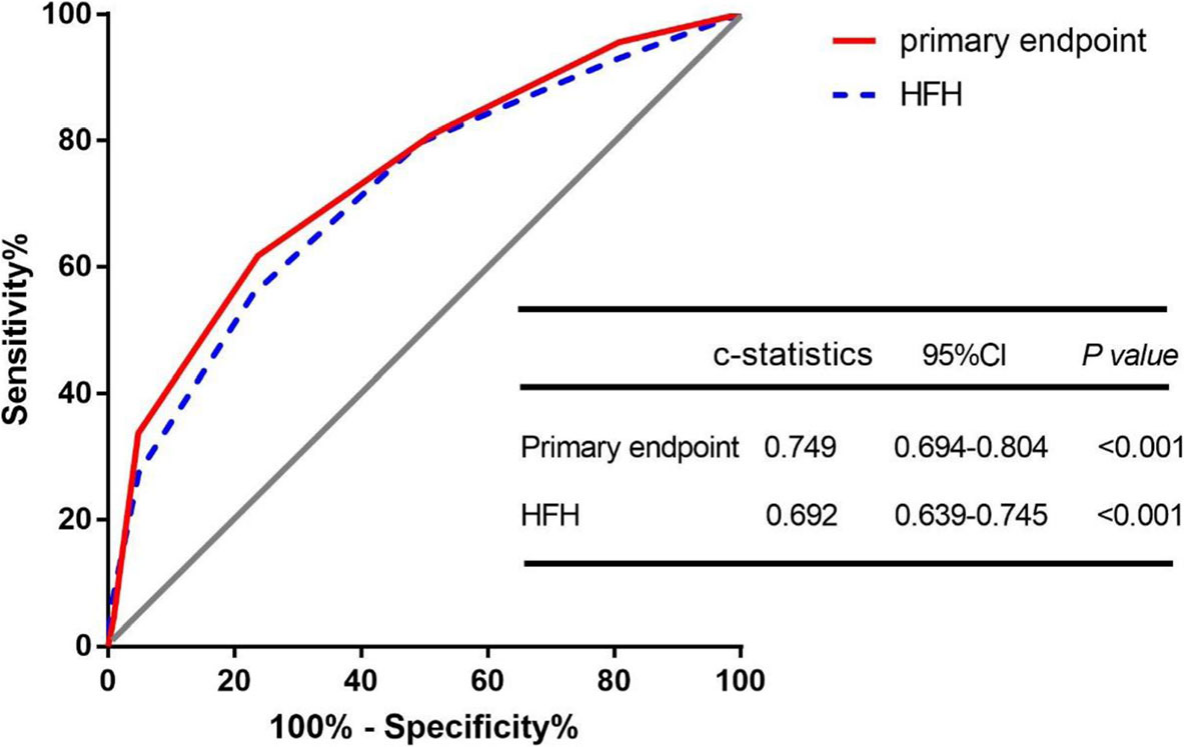
Comparison of area under the curve for Alpha-score of all-cause death and heart transplantation among overall 422 NICM patients with CRT

The c statistics of the model were 0.749 (95% CI: 0.694–0.804, *P* < 0.001) for the primary endpoint and 0.692 (95% CI: 0.639–0.745, *P* < 0.001) for HF hospitalization. (Fig. 4).

## Discussion

### Importance of the new score

This large, observational study first derived a long-term prognosis model for NICM HF patients implanted with CRT. The Alpha-score was based on the largest retrospective cohort of Chinese NICM patients with CRT. The risk score performed well in predicting the long-term prognosis of NICM patients based on clinical characteristics and biomarkers; it showed a good predictive ability for both all-cause mortality and HF hospitalization within the derivation and validation datasets. The Alpha score, as a simple and easy-to-use score, could be used for clinical risk stratification before CRT implantation and long-term follow-up.

### The published scores

Over the past decades, prior risk models [9, 10, 14–16] were derived with good calibration and accuracy in derivation cohorts or Western validation cohorts; however, Asian populations, especially Chinese participants, are rarely used for validation [17]. VALID-CRT [11]and sex category, renal function, ECG/QRS width, ejection fraction, and NYHA class (ScREEN) [8] scores were derived and validated in European multicenter studies, and ejection fraction, atrial fibrillation, age, renal function, and NYHA class (EARRN) [10] does not have a validation population.

The prevalence of ischemic HF in CRT candidates was greater than 50% in most studies [1, 7, 9, 10, 18] conducted in North America and Europe. However, the situation in Asia is significantly different with regards to the subtype of CRT candidates [19–21]. Based on the Japan Cardiac Device Treatment Registry (JCDTR) database [21], the proportion of HF patients with NICM is as great as 70%. Based on our previous studies, patients with NICM were also common (> 60%)in China [6, 22].

Studies have shown that patients with a non-ischemic etiology have a better prognosis than patients with an ischemic etiology. With a lower myocardial fibrosis scar fibrosis scar burden, NICM has the favorable reverse remodeling rather than ICM, which may be a possible mechanism [23, 24].. Thus, various physiological mechanisms may lead to different pathophysiology, clinical status, and response to device therapy. The distinct physiological mechanisms between NICM and ICM could lead to different pathophysiology, clinical status and responses to device therapy. This was a negligible but significant reason for poor discrimination in many of the predictive models among CRT patients. The performance of risk models based on the Western population might be modest in NICM patients with CRT; These scores are readily available to clinicians as they are based on common clinical risk factors, although, it is suggested that recalibration based on different etiologies might improve the applicability of the scores for the NICM population.

### Variables associated with the risk of all-cause mortality and heart failure exacerbation

The five identified baseline covariates in the Alpha score are aligned with those identified in previous studies. Several earlier studies reported that inflammation and heart functional biomarkers were associated with HF outcomes. It is known that inflammation plays an important role in the pathogenesis and progression of heart failure [9, 25]. HsCRP, one of the circulating biomarkers of inflammation related to the severity of heart failure, is a sensitive predictor and is widely used to evaluate clinical outcomes [9, 26, 27]. Chi Cai, et al. [19] indicated that an elevated baseline level of HsCRP level was an independent predictor of adverse survival and increased HF hospitalization. In contrast, other studies [28], with relatively small sample sizes, have shown that baseline levels of HsCRP were not associated with long-term outcomes, and the sample size of those studies is relatively small.

As a marker of ventricle dysfunction, the plasma NT pro-BNP level is a comprehensive index of cardiac function with high sensitivity and specificity. Similarly, in our study, elevated NT pro-BNP levels were an independent predictor of HF progression and mortality, which is consistent with several earlier studies [16, 29, 30].

The LBBB was traditionally a strong predictor of electrical LV discordance in numerous large trials [7, 31–34]. Our results are consistent with the findings of the MADIT-CRT [7], RAFT [35], and REVERSERS [36] trials, which have been shown that non-LBBB patients have an increased rate of mortality due to CRT compared to those with LBBB. A meta-analysis of the major CRT trials also confirmed that a greater benefit from the devices was found when a LBBB morphology was present in patients who underwent CRT, which is strongly recommended for symptomatic HF patients in current guidelines.

The LA, as a reservoir for blood and a contractile chamber, important in LV filling, plays small but significant role in circulation. The size of the LA has been suggested as an independent factor for adverse cardiovascular events in multiple previous studies, especially among HF patients [37–39]. When atrial fibrillation occurs, patients who have undergone CRT not only lose approximately 25% of diastolic blood filling but also suffer from decreased biventricular pacing due to the rapid atrial rate and variability of atrioventricular conduction time. Some studies suggest that the duration of atrial fibrillation, rather than atrial fibrillation itself, is associated with prognosis [31, 40]. In our study, atrial fibrillation included paroxysmal atrial fibrillation and persistent atrial fibrillation. In this study, considering these factors, atrial size is a more reliable indicator of LA function. Although the mechanism remains uncertain, the larger LA size is associated with pulmonary hemodynamic alterations and LA dilatation and dysfunction [7, 34, 39, 41, 42].

Some studies suggest that patient characteristics, such as severity of mitral insufficiency, renal failure, baseline LVEF and age, are risk factors for poor prognosis in patients receiving CRT [9, 16, 42]. However, there are not enough data to prove that these variables are independent predictors in NICM patients.

### Limitations of our study

This study has some limitations. First, as it is an observational, retrospective study. The baseline characteristics were retrieved from Fuwai Hospital’s medical records, and we had no data on serial measurements of biomarkers and ECG parameters. Therefore, our results may not provide model prediction values for the CRT response. Second, the proportion of CRT-D patients in the validation dataset was relatively small, and the patient composition might limit the applicability of the Alpha score in all CRT recipients. Third, data on the final LV lead location, cardiac magnetic resonance imaging for scar tissue and QRS duration after implantation were not collected prospectively. Finally, although the validation dataset was selected randomly in our cohort and determination was good, the potential clinical utility of the Alpha model for risk stratification requires a larger population and further investigation. Despite these limitations, this is the first and largest risk model for NICM patients with CRT in Asia. We believe that the Alpha score could provide useful prognostic information on NICM among CRT recipients.

## Conclusions

In CRT candidates without ICM, **a**trial diameter, non-**L**BBB, **P**ro-BNP, **H**s-CRP, NYH**A** class IV are associated with all-cause mortality and HF transplantation, which could provide a readily available tool for identifying patients who require intensive monitoring and for effective prediction of long-term outcomes among NICM patients who are potential recipients of CRT.

## Data Availability

The datasets used and analyzed during the current study are available from the corresponding author on reasonable request.

## Abbreviations

NICM: Non-ischemic cardiomyopathy
CRT: Cardiac resynchronization therapy
HR: Hazard ratio
CI: Confidence interval
HF: Heart failure
LV: Left ventricle
NYHA: New York heart association
CRT-P: Cardiac resynchronization therapy pacemaker
CRT-D: Cardiac resynchronization therapy defibrillator
ECG: Electrocardiography
NT pro-BNP: N-terminal pro-brain natriuretic peptide
AUC: The area under the receiver-operating characteristic curve
ROC: Receiver operating characteristic
LBBB: Left bundle branch block
LVEF: Left ventricular ejection fraction
ACEI: Angiotensin converting enzyme inhibitors
ARB: Angiotensin receptor blockers
LA: Left atrium
HsCRP: High sensitivity C-reactive protein
JCDTR: Japan cardiac device treatment registry
ICM: Ischemic cardiomyopathy
LDL-C: Low density lipoprotein cholesterol
HDL-C: High-density lipoprotein cholesterol
AST: Aspartate aminotransferase
